# Graphene Oxide–PAMAM Nanocomposite and Ionic Liquid Modified Carbon Paste Electrode: An Efficient Electrochemical Sensor for Simultaneous Determination of Catechol and Resorcinol

**DOI:** 10.3390/diagnostics13040632

**Published:** 2023-02-08

**Authors:** Fariba Garkani Nejad, Hadi Beitollahi, Iran Sheikhshoaie

**Affiliations:** 1 Department of Chemistry, Faculty of Science, Shahid Bahonar University of Kerman, Kerman 7616914111, Iran; 2 Environment Department, Institute of Science and High Technology and Environmental Sciences, Graduate University of Advanced Technology, Kerman 7631818356, Iran

**Keywords:** catechol, resorcinol, carbon paste electrode, graphene oxide–poly(amidoamine) dendrimer nanocomposite, ionic liquid, electrochemical sensor

## Abstract

In this paper, a simple strategy was proposed for the analysis of catechol by a carbon paste electrode (CPE) modified with graphene oxide–third generation of poly(amidoamine) dendrimer (GO/G3–PAMAM) nanocomposite and ionic liquid (IL). The synthesis of GO–PAMAM nanocomposite was confirmed using X-ray diffraction (XRD), energy-dispersive X-ray spectroscopy (EDS), field emission scanning electron microscopy (FE-SEM), and Fourier transform infrared spectroscopy (FT-IR) techniques. The prepared modified electrode (GO–PAMAM/ILCPE) exhibited good performance to detect catechol with a notable decrease in overpotential and increase in current compared with an unmodified CPE. Under optimum experimental conditions, GO–PAMAM/ILCPE electrochemical sensors indicated a lower limit of detection (LOD) of 0.034 μM and a linear response in the concentration range of 0.1 to 200.0 µM for the quantitative measurement of catechol in aqueous solutions. In addition, GO–PAMAM/ILCPE sensor exhibited an ability to simultaneously determine catechol and resorcinol. It can be found that catechol and resorcinol could be completely separated on the GO–PAMAM/ILCPE with the differential pulse voltammetry (DPV) technique. Finally, a GO–PAMAM/ILCPE sensor was utilized to detect catechol and resorcinol in water samples with recoveries of 96.2% to 103.3% and relative standard deviations (RSDs) of less than 1.7%.

## 1. Introduction

Resorcinol and catechol, two isomers of dihydroxybenzene of phenolic compounds, enter the environment because they have a variety of uses in pesticides, cosmetics, antioxidant synthesis, medicines, plastics, dyes, flavoring agents, photography chemicals, rubbers, and other manufacturing industries [1,2]. However, these two isomers are regarded as environmental pollutants and unsafe to humans’ health because of their lower degradation rate and higher toxicity even at very low concentrations [3]. Catechol and resorcinol often coexist together in industrial wastewaters and interfere the detection of one another because of their similar structures and properties [4]. Hence, creating a reliable analytical technique for the simultaneous detection of catechol and resorcinol will be necessary. The available methods that have been reported so far to determine the concentrations of catechol and resorcinol are spectrophotometry [5], gas chromatography coupled with mass spectrometry [6], high-performance liquid chromatography (HPLC) [7], chemiluminescence [8,9], fluorescence [10], and electrochemistry [11,12,13].

Although the majority of these methods are highly accurate, they suffer from complex instrumentation with laborious processes that require higher levels of skill and knowledge. Analytical methods based on electrochemical detection have been attracting attention for many years because of the benefits that are not restricted to throughput and in situ ability, fast response, miniaturization, simplification, inexpensiveness, and biocompatibility [14,15,16,17,18,19]. 

As mentioned in the research, sensing materials crucially contribute to the successful performance of electrochemical sensors so that nanotechnology is one of the major fields to stimulate the construction of high-performance sensors [20,21,22,23,24]. For this reason, experts in the field tended to create, characterize, and assess nanostructured electrodes. The important motivation of the design of nanomaterial-based electrochemical sensors is focused on signal amplification and overvoltage reduction via catalytic activity and conductivity [25,26,27,28,29].

According to the studies, graphene has been introduced as one of the monolayers of sp^2^ hybridized carbon atoms packed into a hexagonal honeycomb lattice. Moreover, graphene oxide (GO) has been considered one of the oxidized derivatives of graphene that is highly hydrophilic with several oxygen-containing groups [30]. On the one hand, researchers have largely considered graphene and its derivatives due to certain physical and chemical features including higher surface area, very good electric conductivity, stronger mechanical strength, easier functionalization, mass production capability, and biocompatibility [31,32,33]. Moreover, GO-based nanocomposites apply synergic effects that increase their performance in electrochemical sensors [34,35]. Dendrimers also have been proposed to be one of the classes of polymers with a major contribution to the creation of nanotechnology [36]. Poly(amidoamine) (PAMAM) dendrimers actually are distinct highly branched, nanoscale macromolecules with multiple active amine groups on the surface. In recent years, researchers have largely considered PAMAM dendrimers due to their chemical stability, their highly geometric symmetry structure, their monodispersity, their globular or ellipsoidal shape, the higher density of the functional groups at the surface, their higher surface area, as well as the higher permeability of the cavities inside them [37,38].

Ionic liquids (ILs) have been described to be liquid-state salts designed and synthesized for certain application via coupling different kinds of anions and cations to fine-tune the features of special ILs [39]. ILs actually enjoy certain physicochemical features such as very low vapor pressure; wide liquid ranges; lower flammability; higher electrical conductivity; suitable solvent features for different kinds of inorganic, organometallic, and organic compounds; higher thermal stability; as well as a big electrochemical window, resulting in their utilization [40,41,42]. In addition, by adjusting ILs’ structure, it is possible to tailor such features for meeting certain utilization requirements [43,44]. However, the most examined dimension of IL-based electrochemical sensors in the electrochemistry field is the use of different types of ILs in modifying electrodes for designing modern electrochemical sensors. Finally, the features exhibited upon the incorporation of ILs into electrodes included greater conductivity, suitable catalytic ability, durable stability (such as stability at higher temperatures), greater selectivity, higher sensitivity, and better linearity [45,46].

Here, we report the modification of a CPE by GO–PAMAM nanocomposite and IL as a sensing platform for the detection of catechol. The electrochemical and electrocatalytic properties of the GO–PAMAM/ILCPE were studied by DPV, cyclic voltammetry (CV), and chronoamperometry (CHA) techniques. Benefiting from the good properties of GO–PAMAM nanocomposite and IL, the obtained GO–PAMAM/ILCPE presented worthy analytical performance for catechol sensing. Moreover, the developed sensor was able to successfully simultaneously measure catechol and resorcinol. Finally, the detection of catechol and resorcinol in water samples was demonstrated by using a GO–PAMAM/ILCPE sensor. The novelty of the present work relates to the role of GO–PAMAM nanocomposite with an excellent catalytic activity that, for the first time, is employed for the detection of catechol and resorcinol.

## 2. Experimental

### 2.1. Laboratory Equipment and Chemicals

Potentiostat–galvanostat (AUTOLAB-PGSTAT 320N, Metrohm, Herisau, Switzerland) was employed to perform all electrochemical measurements. Moreover, data were collected by GPES 4.9 (EcoChemie). We used a Metrohm pH-meter (model: 713, Herisau, Switzerland) supplied with a glass-combined electrode to measure the pH values. Deionized water that was used in each experiment was also taken from Millipore Direct-Q^®^ 8 UV (ultraviolet) (Millipore, Darmstadt, Germany).

Morphological and elemental analyses of prepared material were carried out by MIRA3 SEM (Tescan, Brno, Czech Republic) equipped with an energy-dispersive X-ray spectrometer (EDS) detector. Furthermore, X-ray diffraction (XRD) patterns were acquired through a Panalytical X’Pert Pro X-ray diffractometer (Etten Leur, The Netherlands). FT-IR spectra also were obtained through a Bruker Tensor II spectrometer (Bruker, Mannheim, Germany).

The precursors for the synthesis of GO–PAMAM nanocomposite, catechol, resorcinol, and other chemicals were also of analytical grade and were used without any additional purification. It is noted that they were received from the chemical companies Merck (Darmstadt, Germany).

### 2.2. Synthesis of GO/G3–PAMAM Nanocomposite

The synthesis and characterization of G3–PAMAM were reported in our previous work [47]. The covalence functionalization of G3–PAMAM was performed on the GO surface with 1-ethyl-3-(3-dimethylaminopropyl) carbodiimide hydrochloride (EDC.HCl) and N-hydroxysuccinimide (NHS) as the coupling reactants. For the preparation of GO–PAMAM nanocomposite, ultrasonication of 10 mg GO in 10 mL of deionized water was performed for 2 h in order to reach the best dispersion. In the next step, 40 mg of NHS and 40 mg of EDC.HCl were added to the GO suspension. Then, it was stirred for 40 min at room temperature to activate the GO carboxyl groups. The activated GO was purified by centrifugation, and then deionized water was used to wash it. Then, it was redispersed in deionized water (5 mL). Next, a methanol solution of G3–PAMAM was continuously dropped to the above suspension within 20 min. Next, the mixture was stirred for 3 h at room temperature to accelerate the intended reaction. In the final step, GO–PAMAM nanocomposite was purified.

### 2.3. Preparation of GO–PAMAM/ILCPE Sensor

We prepared the GO–PAMAM/ILCPE with complete manual mixing of graphite powder (0.95 g) and GO–PAMAM nanocomposite (0.05 g) with a ratio of 70*/*30 (% *w/w*) binder *(*paraffin oil*)/*IL *(*1*-*butyl*-*3*-*methylimidazolium hexafluorophosphate) in a mortar using a pestle. After that, we packaged the obtained paste into a glass tube tip, and a copper wire in the paste resulted in an electrical contact. Then, a new surface was recreated via pressing more paste out of the tip and then smoothed by hand via its polishing on the clean paper. Therefore, an unmodified CPE *(*without adding GO–PAMAM nanocomposite and IL*)* was prepared in a similar way for comparison. The surface areas of the GO–PAMAM/ILCPE and the unmodified electrode were obtained by CV using 1.0 mM K_3_Fe(CN)_6_ at diverse scan rates. Using the Randles–Sevcik formula, in the GO–PAMAM/ILCPE, the electrode surface was found to be 0.135 cm^2^, which was approximately 4.3 times greater than that of the unmodified electrode.

### 2.4. Preparation of Water Specimens

Tap water and well water were also sampled, filtrated with a membrane filter, and poured into 0.1 M PBS (pH = 7.0). At last, catechol and resorcinol contents were measured in the water specimens using the as-developed protocol according to a standard addition method.

## 3. Results and Discussion

### 3.1. Characterization of GO–PAMAM Nanocomposite

**Figure 1:**
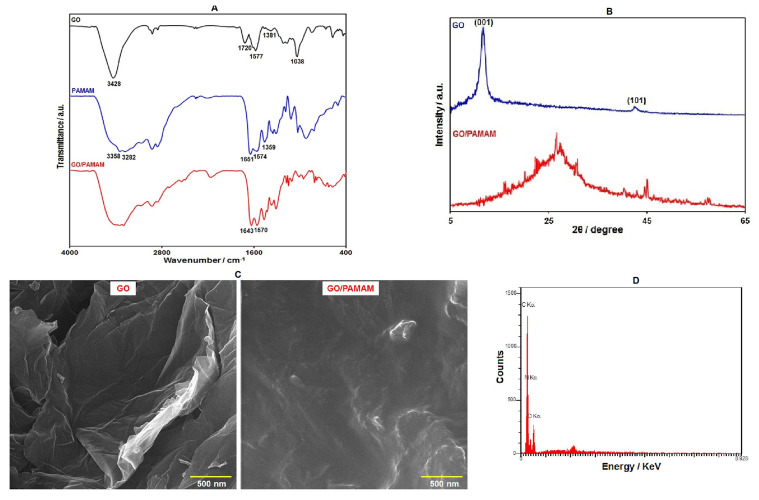
(**A**) FT-IR spectra of GO, G3–PAMAM, and GO–PAMAM nanocomposite, (**B**) XRD patterns of GO and GO–PAMAM nanocomposite, (**C**) FE-SEM images of GO and *GO*–PAMAM nanocomposite, and (**D**) EDS analysis of GO–PAMAM nanocomposite.

The FT-IR spectra for GO, G3–PAMAM, and GO/G3–PAMAM nanocomposite are displayed in Figure 1A. In the FT-IR spectrum of GO, the absorption band in 3428 cm^−1^ was observed, referring to the O–H groups. Absorptions at 1577 cm^−1^ and 1720 cm^−1^ were assigned to the C=C and C=O vibrations, respectively. Other bands were also observed at 1381 cm^−1^ (phenolic C–O) and 1038 cm^−1^ (epoxy C–O–C). Such characteristic bands indicated multiple oxygen-containing functional groups (hydroxyl, epoxy, and carboxyl) on the surface of GO. Considering the FT-IR spectra from G3–PAMAM, the peaks at 3282 cm^−1^ and 3358 cm^−1^ supported the presence of the NH_2_ group. The strong absorption bands at approximately 1651 cm^−1^ and 1574 cm^−1^ corresponded to amides (–CO–NH–) I and II. Furthermore, the absorption band in 1359 cm^−1^ could be related to the C–N stretching vibration. The FT-IR spectra of GO/G3–PAMAM resembles those of PAMAM, with the exception of the complete absence of the stretching vibration of the C=O group at 1720 cm^−1^ in the GO/G3–PAMAM FT-IR case, suggesting a complete reaction of –COOH on the surface of GO with –NH_2_ in PAMAM as well as complete functionalization of PAMAM on GO. We finally observed the characteristic bands of amides (–CO–NH–) I and II in GO–PAMAM nanocomposite at 1643 and 1570 cm^−1^.

Figure 1B shows the XRD patterns for GO and GO/G3–PAMAM nanocomposite. The diffraction peak (001) at 11.5° and a small peak (101) at 42.8° were shown by GO. In the XRD pattern of GO/PAMAM nanocomposite, we found a broad peak at 26.3°*,* which indicated its amorphous nature. However, the diffraction peak of GO disappeared in the XRD pattern of GO–PAMAM nanocomposite, showing an excellent exfoliation and dispersion of GO in the prepared nanocomposite [48].

The structure and morphology of GO and GO–PAMAM nanocomposite were analyzed with FE-SEM images (Figure 1C). The FE-SEM image of GO demonstrates its sheet-like structure. Upon the functionalization of the GO surface with PAMAM dendrimers, a foggy film is observed on the surface of GO sheets. In addition, the GO surfaces are rougher, which reflects the complete attachment of PAMAM dendrimers on the GO surface.

In order to investigate the chemical composition of GO–PAMAM nanocomposite, EDS analysis was performed. The results of the EDS analysis (Figure 1D) revealed the presence of O, N, and C elements in the nanocomposite. C and O elements were mainly derived from GO. In addition, the presence of N in the EDS spectrum confirms GO functionalization with PAMAM dendrimers.

### 3.2. Comparison Study of Electrochemical Reaction of Catechol on Unmodified CPE and GO–PAMAM/ILCPE

**Figure 2:**
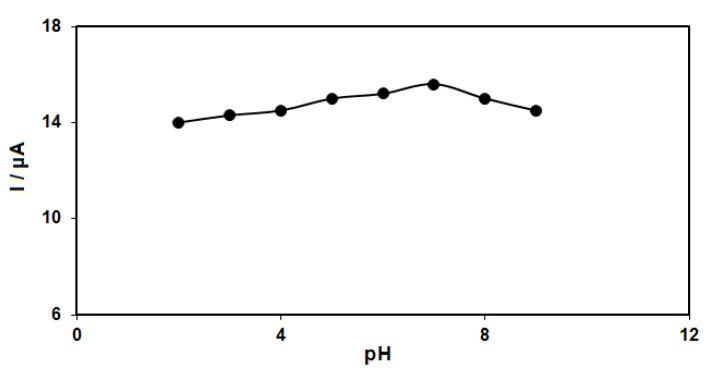
Plot of Ip vs. pH obtained from DPVs of GO–PAMAM/ILCPE in a solution containing 50.0 μM of catechol in 0.1 PBS with different pHs (2.0, 3.0, 4.0, 5.0, 6.0, 7.0, 8.0, and 9.0).

The pH of the buffer solution influences catechol detection. For this reason, the response of catechol (50.0 μM) at the GO–PAMAM/ILCPE was studied in phosphate buffer solution (PBS) (0.1 M) in the pH range between 2.0 and 9.0. The results showed more oxidation of catechol on the surface of the GO–PAMAM/ILCPE in neutral status relative to acidic or alkaline conditions, so pH 7.0 was considered as the optimal value for the electro-oxidation of catechol on the as-produced electrode surface (Figure 2).

**Figure 3:**
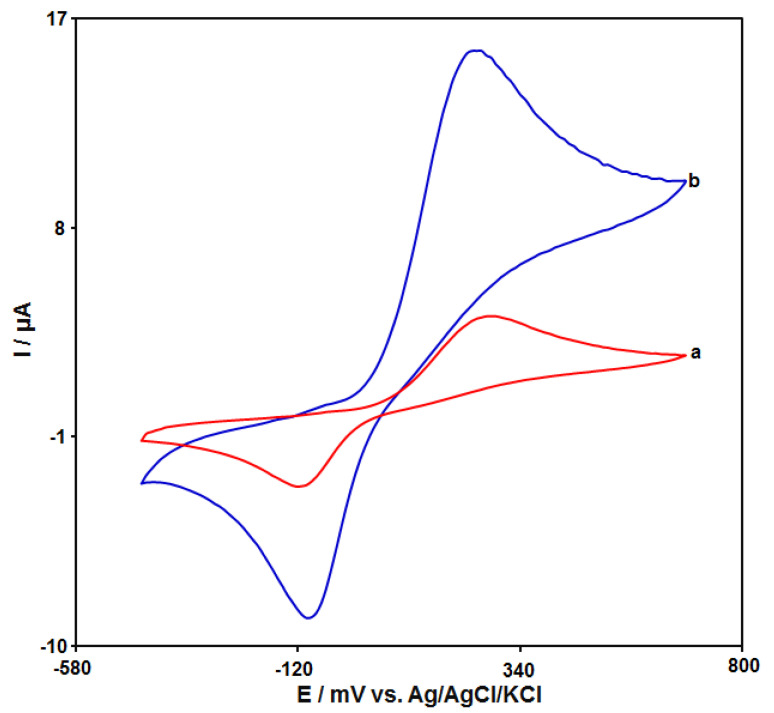
CVs of 50.0 μM catechol at unmodified CPE (curve a) and GO–PAMAM/ILCPE (curve b) in the presence of 0.1 M PBS (pH 7.0) at a scan rate of 0.05 V/s.

The cyclic voltammograms (CVs) of catechol (50.0 μM) on the unmodified CPE and GO–PAMAM/ILCPE in 0.1 M PBS (pH 7.0) at a scan rate of 50 mV/s are shown in Figure 3. Curve a demonstrates the CV of catechol on the unmodified CPE, and weak redox peaks appeared. Compared with the unmodified CPE (in curve a), the GO–PAMAM/ILCPE (curve b) showed the highest response (the anodic peak current (Ipa = 15.6 μA) and the cathodic peak current (Ipc = −8.77 μA)) with the lowest potential (the anodic peak potential (Epa = 250 mV) and the cathodic peak potential (Epc = −90 mV)) toward catechol. Finally, the combination of IL and GO–PAMAM nanocomposite can greatly enhance the detection sensitivity.

### 3.3. Scan Rate Effect on Redox Reaction of Catechol at GO–PAMAM/ILCPE

**Figure 4:**
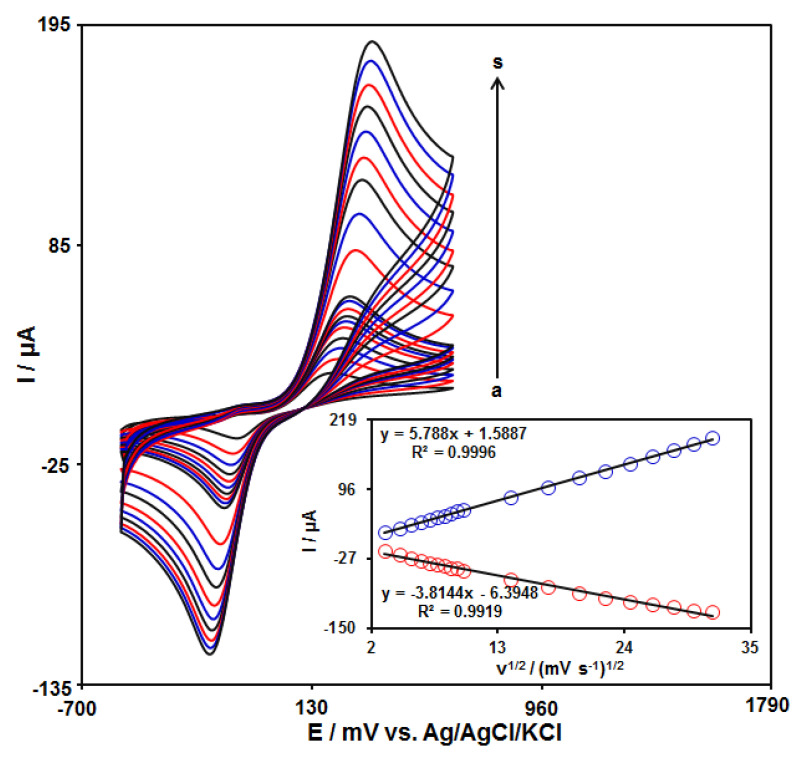
CVs of 150.0 μM catechol at GO–PAMAM/ILCPE in a 0.1 M PBS (pH 7.0) at varying scan rates (a–s refer to 10, 20, 30, 40, 50, 60, 70, 80, 90, 100, 200, 300, 400, 500, 600, 700, 800, 900, and 1000 mVs^−1^). Inset: plot of Ipa and Ipc versus ν^1/2^.

The influence of the scan rate on the redox reaction of catechol at the GO–PAMAM/ILCPE surface was demonstrated using CV at varying scan rates (Figure 4). The voltammograms show an increase in the anodic (Ipa) and cathodic (Ipc) peak currents with enhanced applied scan rates. Moreover, the plot of Ipa and Ipc against the scanning rate square root (*v^1/2^*) exhibited a linear relationship from 10 to 1000 mV/s (Figure 4 (inset)). This specifies that the catechol redox reaction is one of the diffusion-controlled processes.

### 3.4. Chronoamperometric Measurements

**Figure 5:**
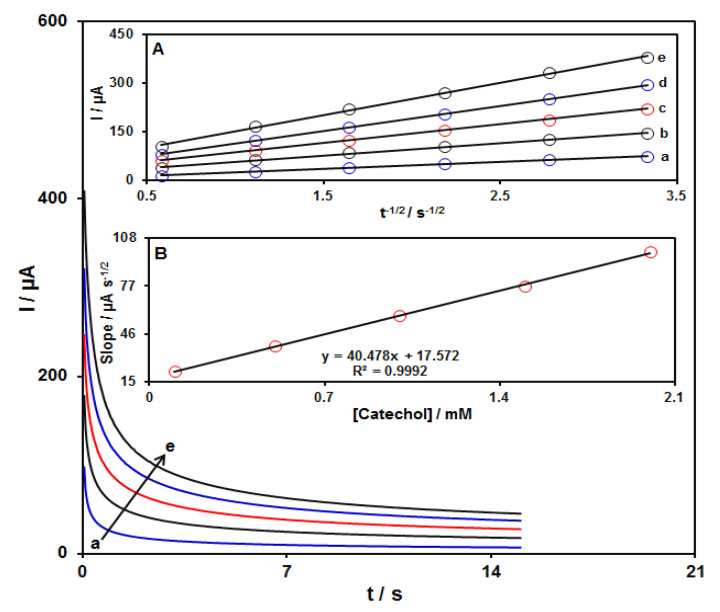
Chronoamperograms obtained on GO–PAMAM/ILCPE in PBS (0.1 M; pH = 7.0) at a potential of 295 mV for variable catechol concentrations (a: 0.1, b: 0.5, c: 1.0, d: 1.5, and e: 2.0 mM). Insets: (**A**) Variations of I vs. t^−1/2^ taken from chronoamperograms and (**B**) plot of corresponding slopes against catechol concentration.

Chronoamperometric measurements for several concentrations of catechol (0.1–2.0 mM) at the GO–PAMAM/ILCPE were performed by adjusting the working electrode potential at 295 mV versus Ag/Ag Cl (KCl 3.0 M) (Figure 5). Utilizing the Cottrell equation, the diffusion coefficient (D) value can be evaluated from plotting I versus t^−1/2^ (see inset A in Figure 5) [49].

I = nFAC(D/πt)^1/2^

where I = current (μA), n = number of electrons, F = Faraday constant (96485 C/mol), A = electrode area (cm^2^), D = diffusion coefficient of the analyte (cm^2^/s), C = bulk concentration of the analyte (mol/cm^3^), and t = time (s). A curve can be obtained as a linear relationship between the slopes obtained from the I relation versus t^−1/2^ and different concentrations of catechol (see Inset B in Figure 5), leading to the calculation of the D value for catechol. The value of D was equal to 1.7 × 10^−5^ cm^2^s^−1^.

### 3.5. DPV Analysis of Catechol

**Figure 6:**
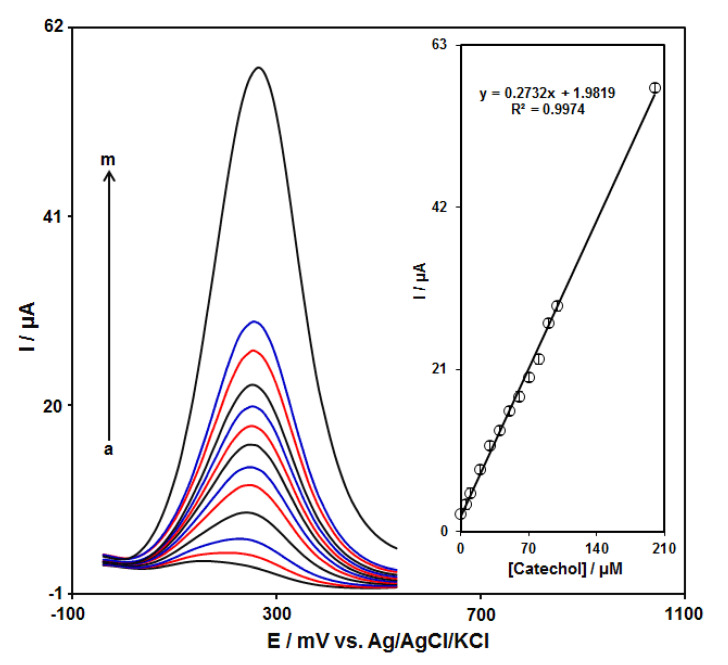
DPV responses of GO–PAMAM/ILCPE sensor toward catechol (a: 0.1, b: 5.0, c: 10.0, d: 20.0, e: 30.0, f: 40.0, g: 50.0, h: 60.0, i: 70.0, j: 80.0, k: 90.0, l: 100.0, and m: 200.0 μM) in a 0.1 M PBS (pH 7.0) at a scan rate of 50 mV/s. Inset: linear correlation between catalytic current and concentration of catechol.

DPV measurements were carried out in solutions containing different catechol concentrations at the GO–PAMAM/ILCPE to obtain a calibration curve (Figure 6). An elevation in the concentration of catechol obviously resulted in an increase in the I_pa_ of catechol. The inset in Figure 6 depicts the association of the current signal with the catechol concentration. As can be seen, it indicates a linear response range for the catechol concentration (from 0.1 to 200.0 μM) with 0.2732 μA/μM sensitivity. The linear regression equation is I_pa_ (μA) = 0.2732C_catechol_ (μM) + 1.9819 with a correlation coefficient of 0.9974. Moreover, the limit of detection, C_m_, of catechol was calculated using the following equation:

C_m_ = 3S_b_/m

where m is the slope of the calibration plot (0.2732 μA/μM), and S_b_ is the standard deviation of the blank response, which is obtained from 10 replicate measurements of the blank solution. The LOD of the prepared sensor is about 0.034 μM. Furthermore, the limit of quantification (LOQ) is about 0.112 μM.

### 3.6. DPV Analysis of Catechol in the Presence of Resorcinol

**Figure 7:**
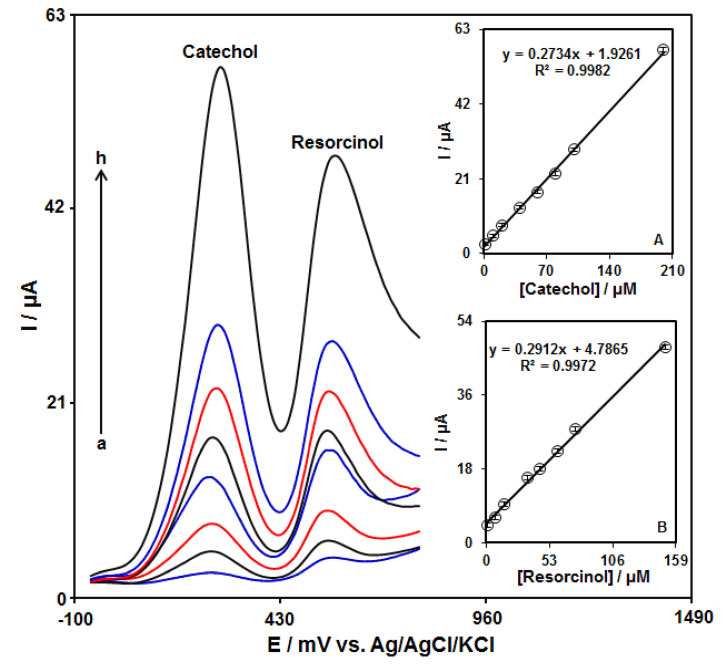
DPV responses of GO–PAMAM/ILCPE sensor in a 0.1 M PBS (pH = 7.0) with various concentrations of catechol (a: 1.0, b: 10.0, c: 20.0, d: 40.0, e: 60.0, f: 80.0, g: 100.0, and h: 200.0 μM) and resorcinol (a: 1.0, b: 7.5, c: 15.0, d: 35.0, e: 45.0, f: 60.0, g: 70.0, and h: 150.0 μM). Insets (**A**,**B**): plot of the correlation of peak current with target concentration.

The simultaneous detection of catechol and resorcinol was studied by using DPV at the GO–PAMAM/ILCPE (Figure 7). After that, two well-separated anodic peaks were considered at 250 mV and 570 mV, which corresponded to the oxidation of catechol and resorcinol, respectively. The potential difference between the two anodic peak potentials of catechol and resorcinol (320 mV) was enough for the simultaneous detection of the concentrations of these compounds. In addition, the peak currents of catechol and resorcinol were linearly correlated with their concentrations (Figure 7A,B).

### 3.7. Selectivity of GO–PAMAM/ILCPE

The selectivity of the prepared sensor (GO–PAMAM/ILCPE) toward potentially interfering species (various ions and organic compounds) for catechol and resorcinol was investigated by using DPV. The tolerance limit was defined as the ratio of the concentration of the interfering species to the analyte, which led to a relative error of less than ±5.0%. It was found that 150-fold of Na^+^, Ca^2+^, Zn^2+^, Mg^2+^, NH_4_^+^, Cl^−^, NO_3_^−^, CO_3_^2-^, and SO_4_^2−^ and 10-fold of phenol, 2-nitrophenol, and 4-nitrophenol did not remarkably interfere catechol and resorcinol determination.

### 3.8. Repeatability and Stability

The stability of the GO–PAMAM/ILCPE was tested by detecting 30.0 μM catechol from 1- to 14-day intervals in PBS (pH = 7.0, 0.1 M). The GO–PAMAM/ILCPE sensor presented that only 3.6% of the current variation was observed with an RSD of 3.4%.

The repeatability of the GO–PAMAM/ILCPE sensor was determined in PBS (pH = 7.0) containing 30 µM catechol. The GO–PAMAM/ILCPE sensor showed exceptional repeatability with an RSD of 4.2% for six repetitive measurements carried out using a single electrode. Hence, it can be concluded from these results that the GO–PAMAM/ILCPE had long-term stability and good repeatability.

### 3.9. Analytical Application

For studying the functional utilization of the GO–PAMAM/ILCPE, we used a standard addition method to analyze the catechol and resorcinol content in water samples. Considering Table 1, recovery of these compounds' detection is 96.2% to 103.3%, reflecting the possible use of the GO–PAMAM/ILCPE sensor for detecting catechol and resorcinol. 

## 4. Conclusions

In the present work, GO–PAMAM nanocomposite and IL were applied for the modification of a CPE to enhance the electrocatalytic activity toward the redox reaction of catechol. Compared with a bare CPE, the synergistic impacts of GO–PAMAM nanocomposite and IL were obvious. By using DPV, the GO–PAMAM/ILCPE sensor linearly responded to catechol within a wide concentration range (0.1–200.0 µM), showing 0.2732 µA.µM^−1^ sensitivity and an LOD of 0.034 μM. In addition, we observed the simultaneous detection of catechol and resorcinol on the modified electrode. On the GO–PAMAM/ILCPE, two distinct peaks corresponding to the oxidation of catechol and resorcinol appeared at 250 mV and 570 mV, respectively. Finally, the practical feasibility of the suggested sensor was shown by catechol and resorcinol detection in water samples.

## Figures and Tables

**Table 1:** Results of determination of catechol and resorcinol in real samples (tap water and well water) using GO–PAMAM/ILCPE.

Sample	Spiked (μM)		Found (μM)		Recovery (%)		R.S.D. (%)	
	Catechol	Resorcinol	Catechol	Resorcinol	Catechol	Resorcinol	Catechol	Resorcinol
Tap Water	0	0	-	-	-	-	-	-
	5.0	4.0	5.1	3.9	102.0	97.5	2.1	3.1
	7.0	6.0	6.8	6.2	97.1	103.3	3.6	2.2
	9.0	8.0	9.1	7.7	101.1	96.2	1.7	2.9
	11.0	10.0	10.9	10.1	99.1	101.0	2.4	1.9
Well Water	0	0	-	-	-	-	-	-
	4.5	5.5	4.6	5.3	102.2	96.4	3.0	2.3
	6.5	7.5	6.4	7.6	98.5	101.3	3.6	1.7
	8.5	9.5	8.6	9.3	101.2	97.9	2.4	3.2
	10.5	11.5	10.4	11.8	99.0	102.6	1.9	2.5

## Data Availability

The data presented in this study are available on request from the corresponding authors.
